# Supplementary material for: Syntrophic anaerobic photosynthesis via direct interspecies electron transfer
Source: Nat Commun. 2017 Jan 9;8:13924. doi: 10.1038/ncomms13924 (PMC5227917; doi:10.1038/ncomms13924)
Supplement: Supplementary Information — Supplementary Notes and Supplementary Figures [file ncomms13924-s1.pdf]

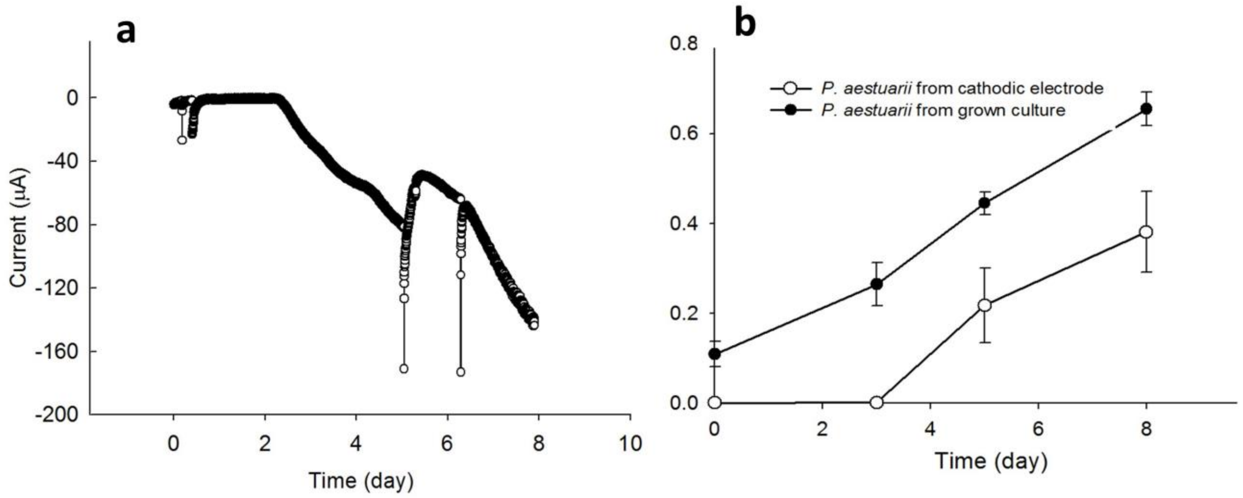

Supplementary Figure 1. *P. aestuarii* uptake electrons directly from electrode for its growth**.**

(a) Medium replacement did not stop electron uptake from the electrode by *P. aestuarii*. Instead, the cathodic current continued to increase, suggesting that mediators were not required for electron uptake by *P. aestuarii.* The decreasing current (from -80 µA to ~-50 µA) after the first exchange may be due to the detachment of some cells that were loosely attached to the electrode. **(b)** The growth of electrode-attached *P. aestuarii* in sulfide-containing medium. Growth observed five days post inoculation confirms their viability on the electrode. The error bars represent the standard error of the mean of replicated experiments (*n*=2).

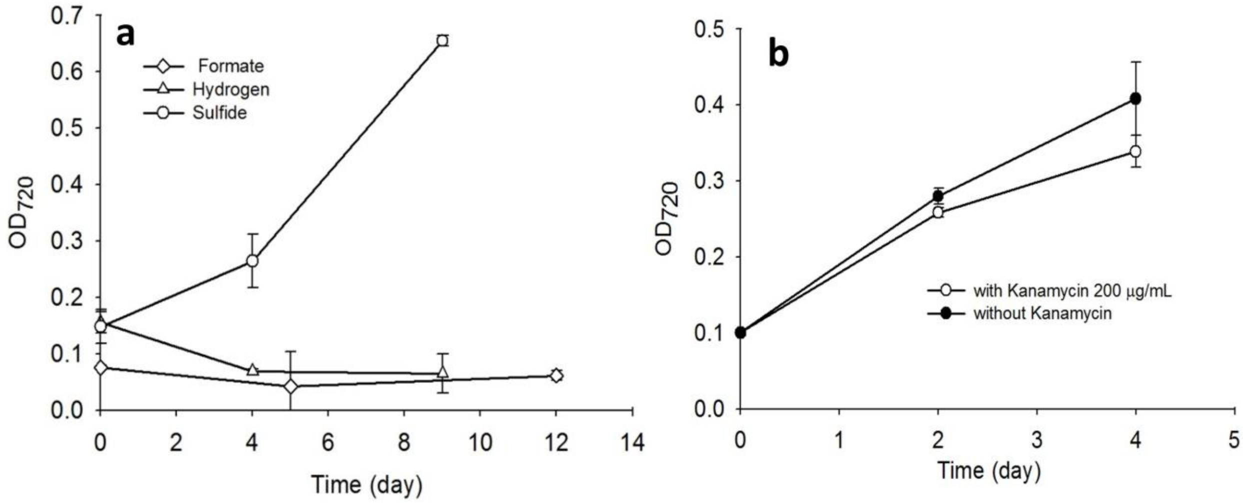

Supplementary Figure 2. Variation of cell intensity of *P. aestuarii* cultures with different substrates and in presence of kanamycin. **(a)** Cell intensity (OD_{720}) of *P. aestuarii* cultures with different substrates as electron donors. *P. aestuarii* does not grow with formate or hydrogen as the sole electron donor but does with sulfide. The error bars represent the standard error of the mean of replicate experiments (*n*=3). **(b)** Growth of *P. aestuarii* in sulfide-containing medium with 200 µg/ml kanamycin. The growth indicates that *P. aestuarii* is not significantly inhibited by kanamycin (at 200 µg/mL), which was added to the co-culture in order to maintain the *ΔombB-omaB-omcB-orfS-ombC-omaC-omcC* mutant of *G. sulfurreducens*. The error bars represent the standard error of the mean of replicate experiments (*n*=2).

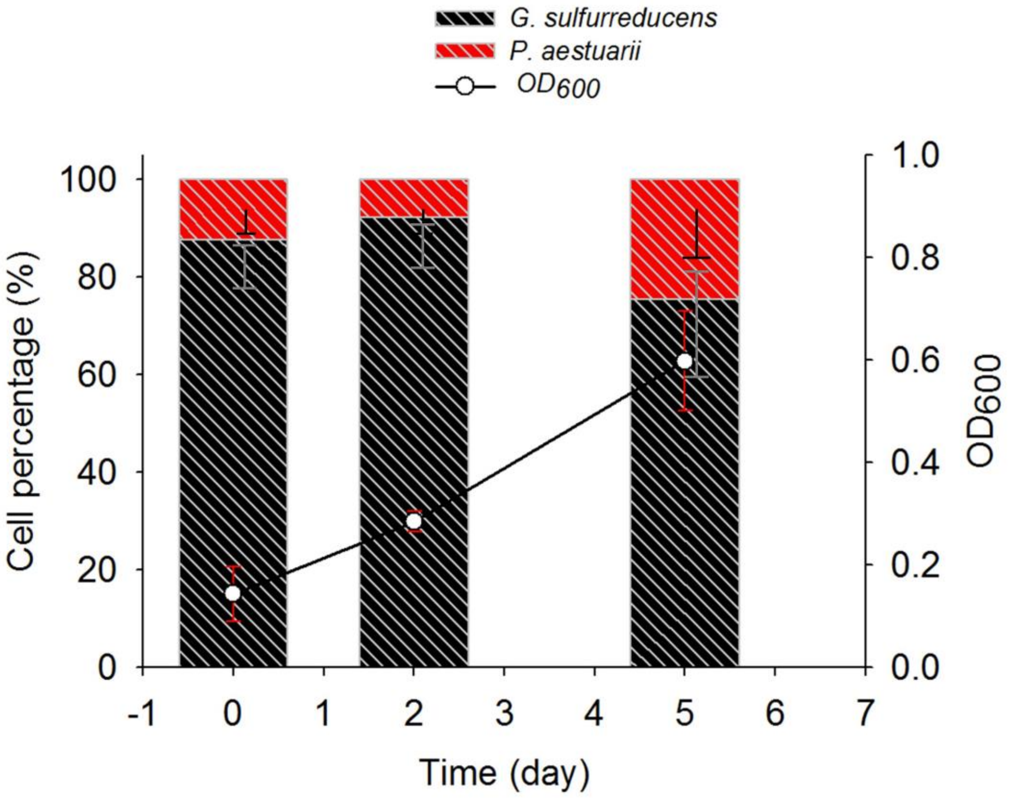

Supplementary Figure 3. Relative abundance of *G. sulfurreducens* and *P. aestuarii* in the co-cultures at various times during their growth. The error bars represent the standard error of the mean of replicated experiments (*n*=3).

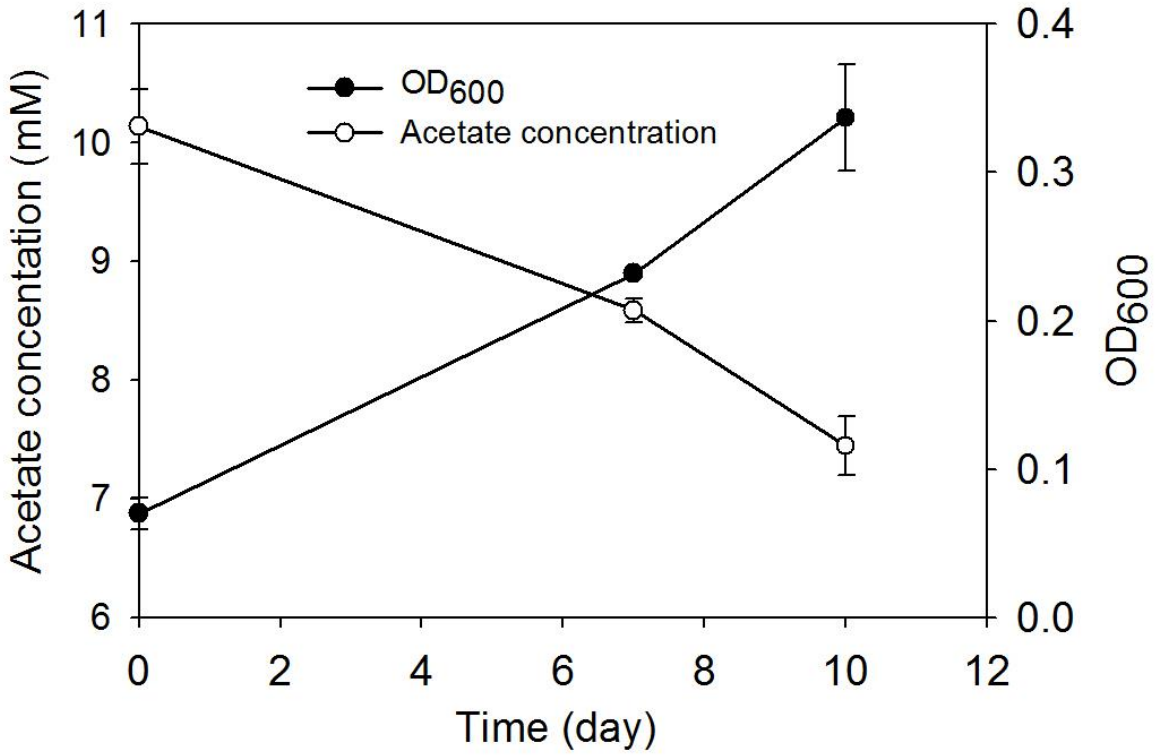

Supplementary Figure 4. Change in acetate concentration and increase in cell density in the transferred co-cultures of *P. aestuarii* and *G. sulfurreducens*. The transferred co-cultures were made by inoculating fresh medium (15 mL) containing acetate as the sole electron donor with 1 mL of previously grown co-culture (OD_{600}~ 0.6). The error bars represent the standard error of the mean of replicate experiments (n=3).

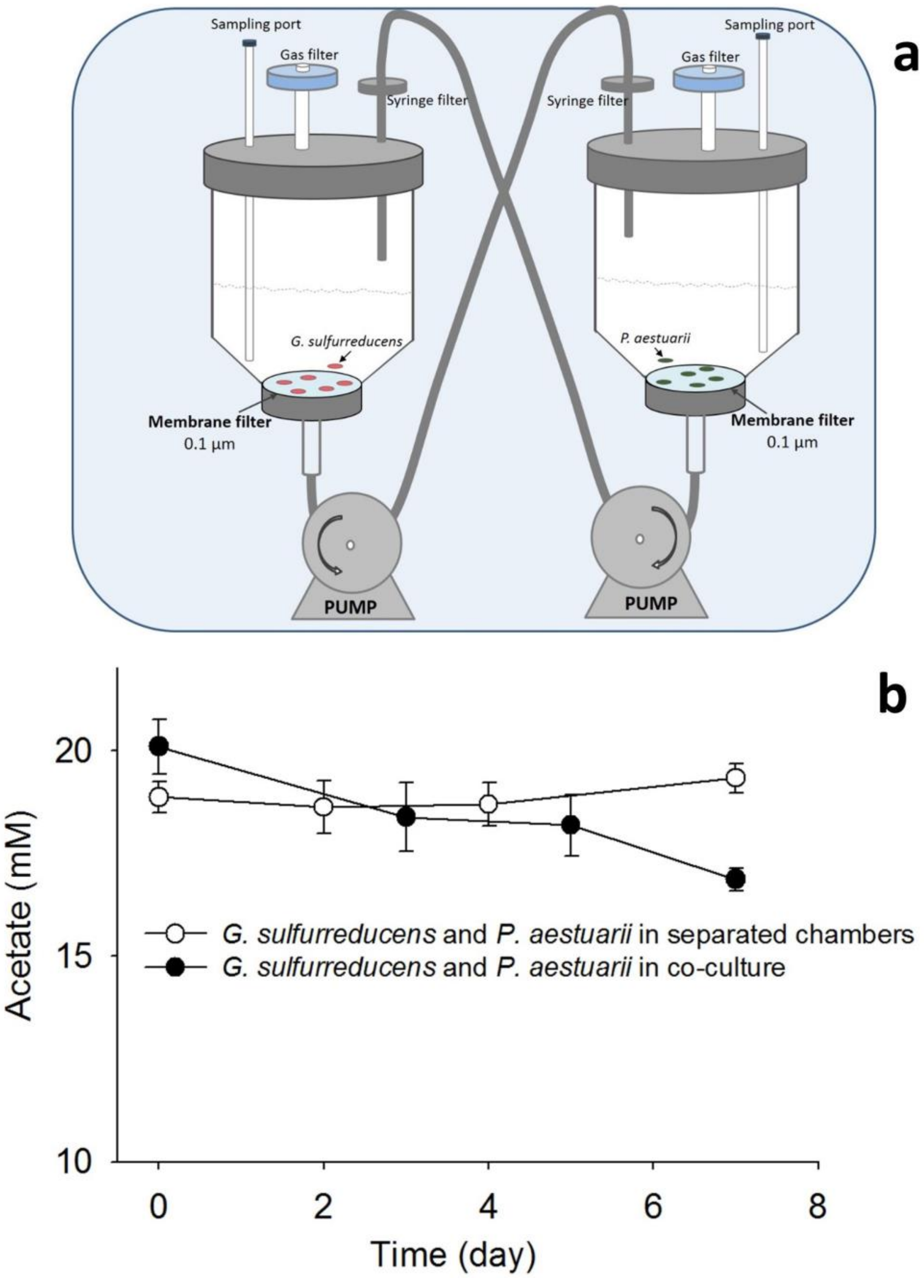

Supplementary Figure 5. Testing the growth of *P. aestuarii* and *G. sulfurreducens* when being cultured in separate chambers while exchanging medium **(a)** Illustrated diagram of growth chambers separated with membrane used to examine whether soluble electron shuttle(s) could be involved in the interspecies electron transfer between *P. aestuarii* and *G. sulfurreducens*. The two species were physically separated into two separated chambers in which filter membranes (0.1-mm pore size) were inserted at the bottom. The effluent of each filter chamber was pumped

(~0.15 ml/min) to another one after being filtered a second time through another filter. **(b)** The total acetate concentration over time when separated chambers were inoculated with the two strains compared with the control experiment (in which a chamber was inoculated with the two strains together). The results suggest interspecies electron transfer between two strains requires direct contact and no soluble electron shuttle(s) is involved. The error bars represent the standard error of the mean of replicate experiments (*n*=3).

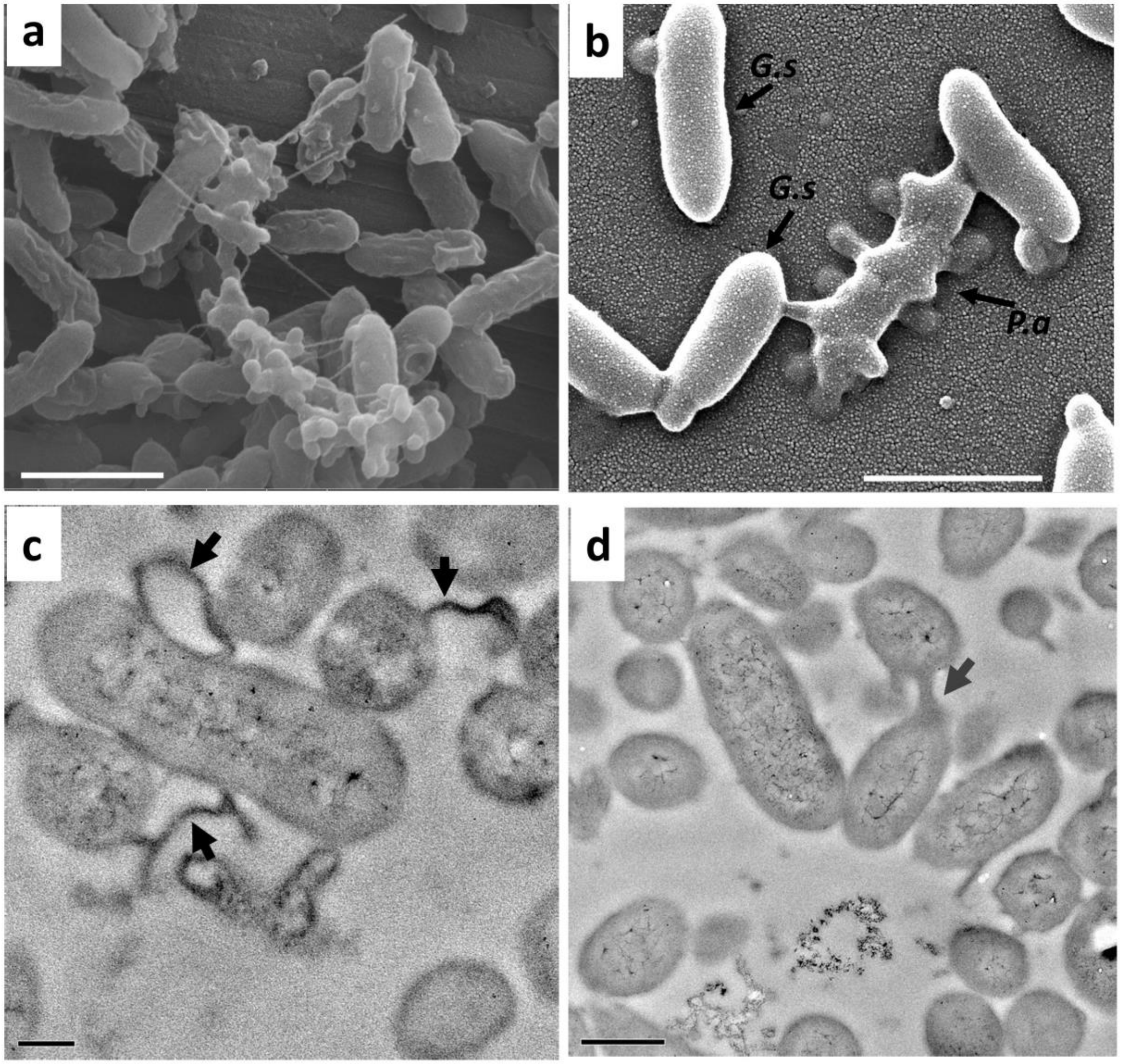

Supplementary Figure 6. EM images of of *P. aestuarii* and *G. sulfurreducens* co-cultures. (**a-b**) SEM images of *P. aestuarii* (*P.a*) and *G. sulfurreducens* (*G.s*) co-cultures display their interconnections via intimate extracellular associations and cell appendages (scale bar, 1µm); these images are representative of 36 recorded images. (**c-d**) TEM images of thin sections of *P. aestuarii* and *G. sulfurreducens* co-cultures treated with heme-reactive compound 3′-3-diaminobenzidine (DAB). (**c**) An abundance of heme-containing pili (black arrows) was found on the positive stained samples which were incubated with DAB and developed with H_2O_2 (scale

bar, 200 nm). This image is representative of 77 recorded images. (**d**) The control samples which were treated with DAB but not developed with H_2O_2 showed filamentous structures (black arrow) with less contrast because of the lack of heme staining (scale bar, 0.5 µm). This image is representative of 17 recorded images.
